# Tuberculosis diagnostics: overcoming ancient challenges with modern solutions

**DOI:** 10.1042/ETLS20200335

**Published:** 2020-12-01

**Authors:** Michael MacGregor-Fairlie, Samuel Wilkinson, Gurdyal S. Besra, Pola Goldberg Oppenheimer

**Affiliations:** 1 School of Chemical Engineering, Advanced Nanomaterials Structures and Applications Laboratories, College of Engineering and Physical Sciences, University of Birmingham, Birmingham B15 2TT, U.K.; 2 Institute of Microbiology and Infection, School of Biosciences, University of Birmingham, Birmingham B15 2TT, U.K.; 3 Healthcare Technologies Institute, Institute of Translational Medicine, Mindelsohn Way, Birmingham B15 2TH, U.K.

**Keywords:** biochemical techniques and resources, diagnostics, tuberculosis

## Abstract

Rapid, sensitive, accurate and portable diagnostics are a mainstay of modern medicine. *Tuberculosis* is a disease that has been with us since time immemorial and, despite the fact that it can be treated and cured, it still remains the world's biggest infectious killer, taking the lives of millions annually. There have been important developments in the diagnostic devices for tuberculosis however, these are often prone to error, expensive, lack the necessary sensitivity or accuracy and, crucially, not sufficiently portable and thus not applicable in the remote, rural areas, where they are most needed. Modern solutions have been emerging in the past decade, seeking to overcome many of the inhibiting issues in this field by utilising recent advances in molecular biology, genetics and sequencing or even completely ‘reinventing the wheel’, by developing novel and unprecedented diagnostic techniques. In this mini review, the issues and challenges arising from the historical methods of diagnosing tuberculosis are discussed, followed by outlaying their particular lack of appropriateness for regions of the world where tuberculosis still remains endemic. Subsequently, more recent developments of new methods and technological advancements as ‘modern weapons’ in the battle to defeat this disease and associated challenges are reviewed, and finally an outlook is presented, highlighting the future of the modern solutions under development, which are envisioned to lay the platform for improvements in delivering timely intervention, reduce immense expense and burden on healthcare systems worldwide, while saving millions of lives and eventually, may enable the eradication of this ancient disease.

## Introduction

Tuberculosis (TB) is an ancient bacterial disease, which is believed to have been infecting humans for over 40 000 years and understood to have originated from the domestication of animals [1]. Its primary causative agent, *Mycobacterium tuberculosis* (*Mtb*), is thought to infect approximately 1 in 3 people worldwide, according to the World Health Organisation (WHO) [2]; accounting for ∼90% of TB infections [3] and as such, this review shall primarily focus on *Mtb*. Other causative agents of TB in humans, include *Mycobacterium bovis* and *Mycobacterium africanum* amongst others and these form the *Mycobacterium tuberculosis* complex (MTBC) [3]. The WHO estimates that TB has claimed the lives of over 1.4 million people in 2019 alone.

Infection establishes itself following inhalation of an exhaled droplet from a patient with an active infection. Upon being inhaled, the bacilli travel to the lungs, where they are engulfed by macrophages, replicate and eventually form a granuloma [4]. At this time point, an infection has established itself within the lungs. For most people (*circa.* 90%) [5], this is where the infection stays; the replication of the bacteria is kept in equilibrium and the infection is latent (Latent Tuberculosis Infection (LTBI)), unable to infect others [6]. For the remaining 10%, due to a litany of factors, the resulting caseous granuloma ruptures and the infection can proliferate and disseminate through the individual's airways to infect other people. In some cases, the bacteria can exfiltrate the lungs and establish an infection elsewhere in the body, causing extrapulmonary TB in ∼15% of cases [2].

Treatment requires a lengthy course of a range of antibiotics. Generally consisting of rifampicin being taken in combination with isoniazid over the course of 6 months, as per recommendations from the National Institute for Health and Care Excellence (N.I.C.E.) [7]. Findings indicate that adverse reactions can be observed in over 75% of patients during rifampicin treatment [8]. Specifically, this can include gastrointestinal disturbances, arthralgia, peripheral neuropathy and drug-induced hepatitis [9]. Additionally, hepatotoxicity can present in up to 28% of patients [10]. Due to these side effects and the duration of the antibiotic course, it is vital to ensure the most appropriate antibiotics are used by correctly identifying drug-sensitive and drug-resistant *Mtb*. This will also bolster patients’ compliance and improve the overall prognosis [11].

Multi-drug resistant TB (MDR-TB)^1^ has been emerging as a major cause for concern. Whilst predominantly emerging from the former USSR [12], apprehensions around the emergence of drug resistance in Africa and Asia are also prevalent amongst the WHO and researchers [13]. It is estimated that MDR-TB was the cause of death in around 182 000 people in 2019 [2,14], with many countries reporting continued rises in the proportion of MDR-TB cases [12] and extensive drug resistant TB (XDR-TB)^2^ also being more frequently reported [15–18]. This is further exacerbated by the co-infection of Human Immunodeficiency Virus (HIV) and TB, particularly in certain areas of South Africa, where up to 36% of the population is known to be infected with HIV [19]. HIV poses a key co-morbidity in TB; being a component in a third of all TB deaths in 2019 [20]. HIV also increases the risk of a latent infection becoming either an active infection or a pulmonary infection becoming extrapulmonary [21].

Diagnosis is typically underpinned by a combined use of molecular detection methods, immunological based assays and direct culture and observation [2]. These methods however, do have known limitations such as, being less effective in individuals infected with HIV (due to their reduced immune response [22]), those with a latent infection, and in children unable to produce significant sputum containing *Mtb*. Furthermore, these methods often require a steady supply of electricity, well-trained personnel and lab modules, which are seldom available in the often under-resourced laboratories in low- and middle-income countries (LMIC) [23]. In terms of diagnosing extrapulmonary TB, diagnosis often remains the same as with pulmonary or latent TB, though it is often combined with the use of invasive biopsies taken from the suspected site of infection [24].

Timely and effective diagnosis for combatting and eradicating TB is at the heart of WHO guidelines [2] with management of latent infections being considered a key component in eradication [25]. However, this goal is poorly supported by current technologies which fall short of the portable diagnostic needs, exhibiting poor-sensitivity, special-handling requirements and complicated, costly procedures. There is, therefore, an urgent need to develop diagnostic methods that can accurately, rapidly, and cheaply diagnose TB particularly, in the rural areas of the Global South.

In this article, current diagnostic methods and their limitations are overviewed. Contemporary technological developments and the future direction of these advancements is further discussed aiming to cover the more recent, novel methods that could be utilised to address the challenges associated with TB in rural areas, such as HIV-TB co-infection, MDR-TB and LTBI.

## ‘State-of-the-art’ diagnostic methods and current challenges

### Direct culture and observation

The ‘gold-standard’ for positive diagnosis of TB is the use of traditional plate culture [26], typically conducted in a high containment laboratory, due to the inherent risk associated with working with *Mtb*. The culturing of *Mtb* raises several issues, mainly relating to the growth rate of *Mtb*, which is substantially slower than other pathogenic organisms, most probably due to a selective pressure at which faster growing *Mycobacterium spp*. induces a greater immune response [27]. It can often take more than four weeks for *Mtb* to produce colonies using traditional Lowenstein–Jensen medium (LJ) [28]. An advantage of plate culture is that drug sensitivity testing can take place concurrently, enabling clinicians to guide antimicrobial therapy more effectively [29].

Mycobacteria growth indicator tubes (MGIT) seek to overcome this using a fluorescent probe, which is quenched by dissolved oxygen within a selective medium. Upon successful cultivation of *Mtb*, the oxygen is depleted, causing the probe to be activated. This is subsequently detected using a light sensor. MGITs have been shown to reduce the mean detection time from 38.6 days to 21.4 days [28]. It is worth noting that to successfully culture the organism requires a high level of expertise and MGIT only offers a rather minor advantage over traditional culture methods [30].

Since*, Mtb* is a group 3 Hazard according to the Advisory Committee on Dangerous Pathogens [31], defined as a biological agent, which ‘*…can cause severe human disease and may be a serious hazard to employees…may spread to the community…'* [31], it requires the corresponding containment facilities to be safely handled. However, this is not always possible within LMICs and, even less so within the more rural settings. Any such facility would also require the relevant expertise and logistics to be run, which is not possible in many of these circumstances [32]. Furthermore, sampling methods of *Mtb* can be inconsistent, with the subsequent sensitivity varying between 16–60% [33]. Sampling methods can involve for instance, a patient producing sputum and ejecting it into a sterile container, or more invasive methods such as, bronchoalveolar lavage and bronchial washing [34]. Both exhibiting a significant difference in their sensitivity of 85.7% *vs* 50.0%, respectively [34]. This can consequently have an impact on the detection rate in local settings, and consideration must also be given to the impact the lavage or washing methods may have on the patients themselves [35].

The use of smear microscopy is often used in tandem with culture, especially in resource poor settings. The method requires the use of the Ziehl-Neelsen stain that dyes *Mtb* and can often result in low sensitivity (56%) in early active cases [36]. Whilst both culture and smear microscopy can be very useful when used together, their efficacy drops significantly in children [37] and HIV or immunocompromised patients due to the lower levels of *Mtb* in the sputum as well as a weakened ability to produce sputum [38,39].

Further methods have been combined with culture to successfully diagnose TB including, the chest X-ray and the Tuberculin skin test [40]. Nevertheless, their efficacy was found to be relatively poor when compared with other more modern methods, which utilise molecular or immunological techniques. The diagnostic flaws of these methods are often attributed to the effect of the human error in interpretation of the results [41]. To overcome this, several companies are developing computational algorithms and the use of Artificial Intelligence (AI) [42] to better interpret chest X-ray results (discussed later).

### Molecular and immunological detection

Historically, the above described direct detection methods have formed the basis of TB diagnostics. However recently, a shift has occurred towards, either molecular or immunological methods. These rely on the detection of molecules or compounds associated with *Mtb* (as opposed to the entire organism) or the detection of components of the immune system reactive to *Mtb* (*e.g.* antibodies). There is currently a growing number of products on the market utilising these approaches. The two most prominent widely used assays are the molecular/Polymerase Chain Reaction (PCR)-based, GeneXpert MTB/RIF® assay and the immunological-based, QuantiFERON-TB gold® assay. GeneXpert first pellets bacteria from sputum and then uses PCR to test for the presence of *rpoB,* a gene in *Mtb* responsible for producing RNA polymerase B. It also tests for resistance to rifampicin, a first-line antibiotic.

Gamma interferon release assays (*e.g.* the QuantiFERON-TB gold®) have been developed to test for TB infection by analysing the levels of gamma interferon released by immune cells (taken from a blood sample) in the presence of TB antigens. If the level of release is found to be above a specified threshold, a patient is considered positive for TB.

There are several additional diagnostic methods for diagnosing TB, though their uptake is not as prevalent [43]. Notably, this includes loop-mediated isothermal amplification (LAMP), which uses an isothermal amplification procedure, in contrast with PCR's multiple, cycling reactions, to produce a result that can be visualised by the naked eye. Although, the sensitivity of LAMP is questionable in smear-negative samples [44] and the reagents required to carry out these tests can often be prohibitively expensive [45], this method is recognised by the WHO [2]. Another example is the line probe assay (LPA), which uses PCR to amplify DNA from *Mtb* and subsequently, applies the output to immobilised oligonucleotides on a strip. These oligonucleotides in turn, emit a colourimetric signal, which signifies the presence of gene mutations conferring drug resistance to isoniazid or rifampicin as well as drug sensitive strains within the sample. LPAs are useful in detecting drug resistance in addition to *Mtb* in smear-positive samples, but are contraindicated in smear negative samples due to their poor detection rate [2,46] as the result of low numbers of individual *Mtb* cells being below the limit of detection. Another WHO approved method is the urine-based lipoarabinomannan assay, which seeks to detect the presence of this *Mtb* compound in the urine of TB patients. These assays have only been indicated in HIV patients and the severely sick^3^ due to overall poor sensitivity in other patient groups [47].

Molecular or immunological approaches can produce results in considerably shorter times than conventional culture. These tests are known to be prone to false positives, and thus a combined use with culture methods enables a more accurate diagnosis. Though limitations become prominent when used in HIV, immunocompromised, paediatric or latent patients [48,49]. Additionally, molecular or immunological assays are costly to deploy and require a good level of expertise and suitable infrastructure to be implemented as evident by their high uptake in high-income countries, such as the United Kingdom yet, almost negligible uptake in countries such as India [50], which often lack such infrastructure.

Overall, the inherent weaknesses of the current diagnostic methods as well as the reasons for their failings are broad and various. Table 1 summarises the methods and their principle with the corresponding benefits as well as drawbacks. Briefly summarising the limitations of the current repertoire of TB tests include being highly time-consuming (in either acquisition or generation of results), requiring specialist laboratory equipment and well-trained personnel, being open to wide interpretation, exhibiting low specificity, which is further exacerbated in HIV and immunocompromised patients as well as children, or infrastructure issues within LMICs. Therefore, recent developments have been sought to overcome many of these limitations by both reducing the equipment and infrastructural requirements and by utilising different sample types.

**Table 1:** Currently used diagnostic techniques, recognised by the WHO, with the associated strengths and weaknesses

Diagnostic method	Sensitivity (95% CI)^4^	Specificity (95% CI)	Strengths	Weaknesses
Bacterial culture	100%^1^	100%^1^	+ Gold standard of sensitivity and specificity + Drug sensitivity testing can take place in tandem (through use of drug diffusion disks) + Cheaper than molecular/immunological methods	− Requires high level biosafety containment laboratory − Generation of results is time-consuming (>28 days)
MGIT (Becton Dickinson and Company, U.S.A.)	86–93%^2^	99.99%^2^	+ Faster than conventional culture + High degree of specificity and sensitivity	− More expensive than conventional culture − Requires specialist training − Method is labour intensive − Requires high-containment lab
Smear microscopy	60–69%^3^	97–98%^3^	+ Rapid, on-site delivery + Cheap + Few reagents are required	− Requires training − Reagents are toxic (e.g. phenol)
Chest X-ray	73–79%^4^	60–63%^4^	+ Readily available in healthcare settings + Non-invasive	− Low specificity & sensitivity − High initial costs, although has multiple applications
Tuberculin skin test (Sanofi, France)	48–78%^5^	57–81%^5^	+ Cheap + Can be used for LTBI + Can be rapidly deployed + Requires no handling of *Mtb*	− Results take ∼5 days to appear − Requires repeated visits to healthcare professional − Highly variable sensitivity and specificity
GeneXpert MTB/RIF (Cepheid, U.S.A.)	82–88%^6^	96–98%^6^	+ Can test for *Mtb* and rifampicin resistance + Rapid turnaround (<2 h) + Cartridges can be stored at room temperature	− High initial start-up cost (>$10 000) − Variable sensitivity in HIV/Immunocompromised patients (73–87%) − Low sensitivity in smear-negative patients (64–75%)
QuantiFERON-TB Gold (Qiagen, U.S.A.)	61–86%^5^	57–81%^5^	+ Can be used for LTBI + Blood sample (which is easier to acquire than a sputum sample) + Rapid detection	− Less sensitive in HIV/Immunocompromised individuals (43–49%) − Less sensitive in children (70–76%) − Requires handling of blood samples − Requires specialist training − Relatively expensive
TB LAMP (Eiken, Japan)	85.6–92.6%^7^	91.0–96.1%^7^	+ Sensitivity and specificity comparable to PCR testing + Cheaper to run than PCR + Visual readout + Rapid detection	− Infrastructure required can be prohibitively expensive − Cannot be used for LTBI − Presents a significant contamination risk if run in a molecular biology laboratory
Line probe assays for *Mtb* (Hain Lifescience, Germany & Nipro, Japan)	95.6–97.5%^8^	98.7–99.5%^8^	+ Can detect resistance to isoniazid and/or rifampicin + Rapid detection (<6 h)	− Less sensitive and specific in smear-negative samples (62.5–74%) and (84.9–92.8%) − Requires electricity − Reagents require refrigeration/freezing − Cannot be used for LTBI
Determine TB LAM Ag test (Abbott, U.S.A.)	13–93%^9^	87–99%^9^	− Non-invasive − Rapid detection − Useful in immunocompromised/HIV patients who are seriously ill^#^	− Large variability in sensitivity − Not recommended in immunocompetent individuals

^#^Defined as *‘respiratory rate >30 breaths per min, body temperature >39°C, heart rate >120 beats per min, or unable to walk unaided'*

1 Nambiar R, Bereksi N, Gonzalez R, de Cozar A, Loubet M, Shetty A, et al. Performance of bioMérieux Lowenstein–Jensen slopes in plastic tube packaging, compared to existing phenotypic methods, for efficient recovery of the Mycobacterium tuberculosis complex. Journal of Medical Microbiology. 2019;68(3):398-401;

2 Cruciani M, Scarparo C, Malena M, Bosco O, Serpelloni G, Mengoli C. Meta-analysis of BACTEC MGIT 960 and BACTEC 460 TB, with or without solid media, for detection of mycobacteria. J Clin Microbiol. 2004;42(5):2321-5;

3 Davis JL, Cattamanchi A, Cuevas LE, Hopewell PC, Steingart KR. Diagnostic accuracy of same-day microscopy versus standard microscopy for pulmonary tuberculosis: a systematic review and meta-analysis. The Lancet Infectious Diseases. 2013;13(2):147-54;

4 Piccazzo R, Paparo F, Garlaschi G. Diagnostic Accuracy of Chest Radiography for the Diagnosis of Tuberculosis (TB) and Its Role in the Detection of Latent TB Infection: a Systematic Review. The Journal of Rheumatology. 2014;91:32-40;

5 De Keyser E, De Keyser F, De Baets F. Tuberculin skin test versus interferon-gamma release assays for the diagnosis of tuberculosis infection. Acta Clinica Belgica. 2014;69(5):358-66;

6 Li S, Liu B, Peng M, Chen M, Yin W, Tang H, et al. Diagnostic accuracy of Xpert MTB/RIF for tuberculosis detection in different regions with different endemic burden: A systematic review and meta-analysis. PLOS ONE. 2017;12(7):e0180725;

7 Nagai K, Horita N, Yamamoto M, Tsukahara T, Nagakura H, Tashiro K, et al. Diagnostic test accuracy of loop-mediated isothermal amplification assay for Mycobacterium tuberculosis: systematic review and meta-analysis. Sci Rep. 2016;6(1):39090;

8 Nathavitharana RR, Cudahy PGT, Schumacher SG, Steingart KR, Pai M, Denkinger CM. Accuracy of line probe assays for the diagnosis of pulmonary and multidrug-resistant tuberculosis: a systematic review and meta-analysis. European Respiratory Journal. 2017;49(1):1601075;

9 Minion J, Leung E, Talbot E, Dheda K, Pai M, Menzies D. Diagnosing tuberculosis with urine lipoarabinomannan: systematic review and meta-analysis. European Respiratory Journal. 2011;38(6):1398-405.

## Recent developments

### ddPCR

More recent developments have started utilising some of the newly emerging technologies such as, digital droplet PCR (ddPCR). ddPCR is a more recent innovation which partitions the amplification reaction seen in PCR; this way, it provides an absolute quantification of gene expression rather than a relative one. Due to this, its sensitivity is higher than qPCR, and this method is capable of detecting single copies of DNA [51]. This technique has already been used to monitor mixed populations of cells to better understand drug-resistance [52]. Whilst ddPCR offers a greater amount of sensitivity than quantitative or qPCR methods and can be used to test for *Mtb* infection in both sputum and blood samples [53,54], and therefore useful in cases of pulmonary, extrapulmonary, LTBI and active TB, though its main drawback is that it can be prohibitively expensive [55].

### CRISPR

**Figure 1.**
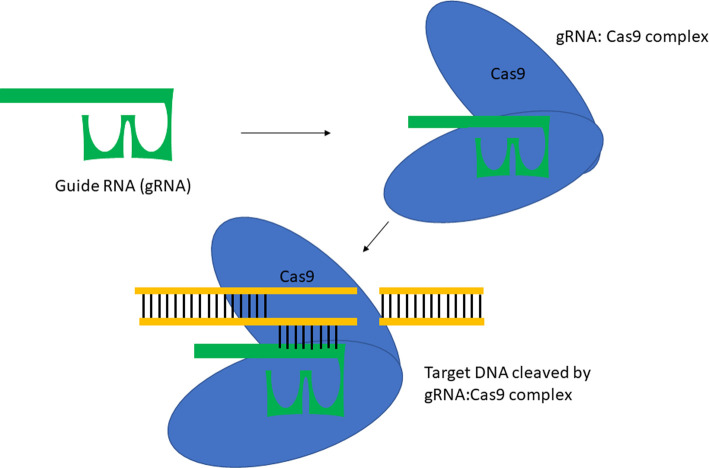
Representation of the CRISPR/Cas9 system. The guide RNA (gRNA) forms a complex with Cas9 to mediate cleavage of the target DNA sites that are complimentary to the gRNA.

**Figure 2.**
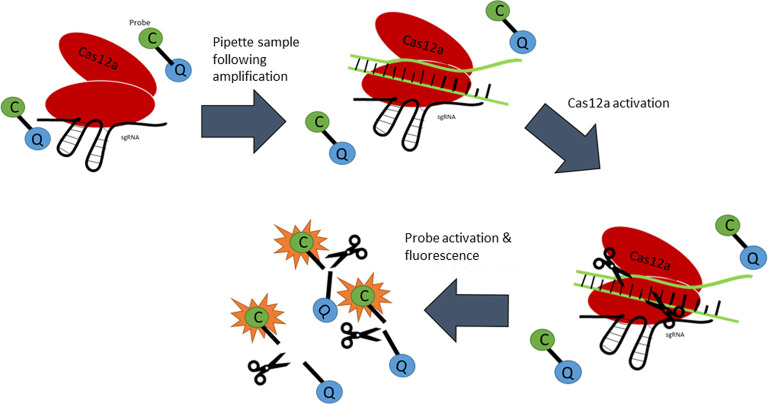
Schematic representation of the CRISPR/Cas12a system. The single-strand guide RNA (sgRNA) forms a complex with Cas12a whilst fluorescent probes (C) are in the periphery, bound to quenchers (Q) with an oligonucleotide. The target DNA enters the complex and is cleaved by the Cas12a enzyme. The enzyme subsequently cleaves the probes and activates the fluorescent signals through its peripheral action.

**C**lustered **R**egularly **I**nter**SP**aced **R**epeats or CRISPR and its combined use with **C**RISPR **AS**sociated nuclease 9, Cas9 (Figure 1) is a technology that is often thought of as a gene-editing technique. Essentially, it has acted as a programmable enzyme that can ‘cut' DNA in specific places, though recent discoveries have identified more enzymes which possess collateral activity as well as an ability to target single-stranded DNA, as is the case with Cas12a [56]. Contemporary research has demonstrated that this technique could be used to detect *Mtb* DNA [57]. This is particularly noteworthy due to its potential to be lyophilised onto a lateral flow stick with attomolar sensitivity, enabling it to be readily deployed in areas where electricity is seldom available [58]. Ai et al. have utilised a recombinase polymerase amplification (RPA) prior to placing onto the lateral flow stick. RPA is a DNA/RNA amplification technique that can be run at a relatively low temperature (*ca.* 37°C). The speed of the amplification of RPA is proportional to the temperature (e.g. the higher the temperature, the faster the reaction) as it amplifies IS6110. One of the key advantages of CRISPR based diagnostics is that it can be rapidly developed and tailored to quickly identify novel mechanisms of drug-resistance [59], allowing it to supersede PCR-based methods. Adding to this, is the ability to be used in tandem with PCR to significantly bolster the limit of detection in more resource-rich settings [60]. Furthermore, it has been demonstrated that the technique has a better overall diagnostic performance when compared with the GeneXpert MTB/RIF® assay as well as a faster turnaround [57]. The overall principle of this technique is summarised in Figure 2.

### Next-generation sequencing techniques

Another approach to the diagnosis and surveillance of *Mtb* is to utilise next-generation sequencing (NGS) technology to sequence *Mtb* genomes allowing diagnosis, screening of these genomes for resistance conferring mutations, and determination of their relatedness for public health purposes. Drug sensitivity testing (DST) can be achieved either via targeted or by whole-genome sequencing (WGS) approaches. Targeted NGS approaches, such as, the Deeplex®-MycTB assay [61], are focussed on known resistance conferring loci, whereas WGS is focused on sequencing the entire genome [62]. Currently, targeted approaches are the most attractive for rapid DST in a clinical context due to the greater reliability and availability of commercial assays [61]. The major problem with this approach is its inherent lack of flexibility, considering it is reliant on characterised resistance-conferring mutations when the understanding of the genetic basis of antibiotic resistance phenotypes is far from complete and there is significant evidence that genome-wide changes contribute to resistance phenotypes [63]. WGS offers several advantages the foremost being its inherent flexibility and the accuracy of predictions can be improved iteratively due to better models being developed. A genomic epidemiological approach, where *Mtb* transmission clusters are identified phylogenetically allowing evidence-based interventions to be utilised to control *Mtb* transmission more effectively on a population-wide scale can only effectively be achieved with WGS. The epidemiological applications of NGS are particularly intriguing for public health. The ability to track clusters and larger population-wide trends in real-time could revolutionise our approach to *Mtb* as a public health problem. Machine learning models of antibiotic resistance prediction utilising WGS data have been shown to outperform a targeted approach significantly for first- and second-line drugs and will only improve further as more complete phenotypically characterised *Mtb* genomes are becoming available. Previously, the major barriers for the utilisation of WGS in rapid *Mtb* DST were the difficulties associated with directly sequencing whole *Mtb* genomes from clinical samples without a culture step. The small quantity of *Mtb* genetic material within sputum compared with DNA from the patient has been a significant hurdle and even successful protocols capable of producing high quality genomes directly from sputum [64] have not been widely adopted due to the high reagent costs. However, a recent protocol seems to present a viable strategy to selectively enrich clinical samples for *Mtb* without the use of costly reagents. By utilising a thermo-protection buffer, which mimics the conditions within hyperthermophiles, patient's DNA was selectively degraded during heat inactivation of *Mtb,* a necessary step due to *Mtb* being a category III Hazard, requiring a lab, which conforms to Level III containment. Without further studies however, it is not possible to categorically state the impact of this work yet, although the demonstrated results appear very promising. Further consideration regarding the viability of WGS approaches must be given to which sequencing platform should be utilised. Illumina-based platforms are widely used for healthcare sequencing applications due to their low cost per sample and high accuracy. However, in low- and middle-income countries, where *Mtb* carriage is most common, the initial costs and infrastructural requirements of Illumina-based platforms are a significant barrier to adoption [64]. Oxford Nanopore Technologies have introduced the possible alternative of nanopore sequencers, which have low initial costs and significantly more achievable infrastructural requirements. However, the cost per-sample of these sequencers is significantly higher, and their marginally lower per-base accuracy may reduce their utility for determination of resistance mutations. This device has been previously employed in Liberia, Guinea and Sierra Leone during the 2014-16 Ebola outbreaks [65]. Whilst NGS and molecular methods such as ddPCR and CRISPR are useful in analysing sputum samples and can enable healthcare workers to rapidly diagnose individuals, as well as identify patients with antibiotic resistant infections, the above described limitations exist with immunocompromised and paediatric patients due to the lack of a viable sputum production.

### MicroRNA detection

Concurrently, Cui et al. have been exploring the use of a PCR based assay, which seeks to identify microRNAs (miRNA) present within the bloodstream of patients [66]. Although the complete capabilities of such an assay are yet to be established, there is a growing interest in identifying relevant miRNAs, which can be used as a diagnostic marker for the previously ‘hard-to-diagnose’ groups such as, paediatric [67] and HIV patients [68]. A dynamic assay would further enable identification of both latent and active TB patients, in addition to suspected extrapulmonary patients. miRNA as a biomarker holds a great potential, however substantial lab equipment and a centrifuge would be required to carry out this assay, representing a key-drawback for its exploitation for this purpose. Togun et al. have highlighted the need for the correct miRNA sequences to be identified in order to ensure the efficacy of such a test; they have also shown that an extensive number of micro-RNAs are found in childhood TB cases of which, only 7% are considered feasible in the proposed assay [69].

### eNose

Alternative developments include a portable, handheld device, which can provide a diagnosis by analysing the breath of suspected TB patients *via* volatile organic compounds (VOC), indicative of an infection. The Aeonose (eNose BV, Zutphen, Netherlands) constitutes an example of such device and it has been tested in the field [70]. Preliminary results have so far fared relatively poorly [71], however the portability and the rapid generation time of results (<5 min) makes the prospect of this technology highly appealing particularly, in rural areas.

### Raman spectroscopy

Raman spectroscopy (RS) is another portable analytical tool, which could be readily deployed into rural areas. RS has been utilised in many other applications, such as the diagnosis of cancer and to identify bacterial infections [72]. The technique utilises the phenomenon of Raman scattering to detect the unique molecular fingerprints of various bacteria when subjected to an excitation with a certain wavelength of light. Preliminary research has demonstrated its ability to detect TB alone (both drug-sensitive and drug-resistant serotypes) [73–75], however no research exists as to its efficacy outside of a monoculture sample (a critical objective if such a device were to make it to market) or alternative samples to blood. The successful development of an RS based method would enable clinicians to rapidly screen through a vast number of patients due to Raman's ability to scan a sample within a matter of minutes, as opposed to many hours other molecular methods use.

### AI processing

As described above, AI processing is becoming increasingly relevant for the analysis of historical methods of detection of TB such as, the chest X-rays [42] and, it is also being used to process micrographs from smear microscopy [76]. By utilising AI, human error in the interpretation of results can be significantly diminished, with previous research demonstrating that AI is superior to humans within this context [42].

Limitations persist in that the technology is still within its initial stages and it has yet to be tested using a large sample size. Furthermore, the technology itself has been shown to vary wildly pending upon the population it is being used in *e.g.* variance between HIV co-infected patients, paediatric populations, *etc.* [77]. Regardless, technique is currently being developed by several companies in the world and is under consideration by the WHO as a technique that can help combat TB [2]. Another benefit of the technique is that images could be sent from rural areas to a server site for analysis with the AI *via* the mobile phone technology [78].

### Graphene-based biosensors

Lastly, Eloi et al. describes the use of a biosensor containing an arginine film which, when combined with *Mtb* based probes is able to gauge the variance in electrochemical measurements following hybridisation [79]. A similar method using a graphene-based sensor has also been identified, with nearly 100% accuracy [80]. These biosensors require DNA extraction prior to their use however, both the extraction and hybridisation steps can take place at room temperature; thus allowing deployment in rural areas with the use of a portable power supply. Whilst biosensors are promising, numerous issues persist; specifically, in relation to their sensitivity. Limits of detection are described as 10^3^ copies of DNA [80], which in a stark contrast with the techniques identified by Ai et al. and ddPCR, where only a single copy is necessary. With these, there are also issues in identifying latent, immunocompromised, HIV and paediatric patients due to their inability to produce sputum as readily. Nevertheless, it represents yet another PCR-free alternative for TB diagnostics.

Overall, there are several promising techniques for TB diagnostics currently being researched and developed around the world, many of which have the potential to supersede the existing repertoire of diagnostic tests, in the near future. Whilst the majority of these are not likely to resolve all of the previously identified challenges, their inherent improvements and advantages lay the platform to overcome many of the current issues and, a combined use of these tests could help considerably to overcome the burden of TB, enable diagnosing patients earlier and, provide a better treatment, management and improved prognosis in the longer term. The discussed techniques are summarised in Table 2.

**Table 2:** Summary of the discussed TB diagnostic methods and their corresponding benefits and drawbacks

Diagnostic method	Reported sensitivity	Reported specificity	Strengths	Weaknesses
ddPCR^1^	NA^i^	NA	+ Reported sensitivity down to 1 copy of DNA per sample + Provides absolute quantification of sample + Flexibility to analyse sputum and blood	− Prohibitively expensive ($10,000+) − Requires uninterruptible power supply
CRISPR/Cas12a	90%^2^	98%^2^	+ Rapid turnaround (<1.5 h) + High sensitivity and specificity + Reaction can take place at room temperature + Reaction can be readily visualised + Reaction can be readily adapted to cover other serovars of *Mtb* and drug-resistant strains + Culture-free + Can use multiple sample sites	− Non-specific targeting is feasible − Efficacy not established for paediatric or HIV/Immunocompromised patients
Deeplex-MycTB (Genoscreen, France)	NA	NA	+ Detection of Rifampicin resistance supersedes GeneXpert MTB/RIF^3^ + Provides resistance profiles to 15 drugs^4^ + Can be multiplexed to analyse up to 384 samples^4^ + Identifies more than 100 different mycobacterial species^4^ + Culture-free	− Longer turnaround compared with other methods (48 h) − Requires storage at −20°C − Requires PCR to be performed before sequencing − Requires access to web application − Requires molecular biology lab − Requires expensive sequencing equipment − May miss novel sequences conferring resistance − Requires sputum
Whole Genome Sequencing (WGS)	>95%^5^	>95%^5^	+ Detects drug resistance + Whilst price is high, it is continuously decreasing + Enables global surveillance of strains	− Longer turnaround compared with other methods (up to 72 h) − Some techniques are prohibitively expensive (>$95 per sample) − Requires uninterruptible power supply − Requires sputum − May miss novel sequences conferring resistance
MinION Nanopore sequencing (Oxford Nanpore technologies, U.K.)	94.8%^6^	98%^6^	+ Portable + Can detect drug resistance + Cheaper than WGS (<$71 per sample) + Has been deployed in the field^7^ + Quick turnaround (<6 h)	− Requires uninterruptible power supply − High initial start-up costs − Full characterisation to take place − Requires sputum
miRNA	24.7–39.9%^8^ (95% CI)^ii^	>90%^8^	+ Culture-free + Quick turnaround (<24 h) + Blood sample	− High variability in results − Effective miRNA yet to be uncovered − Must be utilised regularly
Aeonose (eNose BV, Netherlands)	75–92%^9^ (95% CI)	44–65%^9^ (95% CI)	+ Non-invasive + Highly portable + Rapid turnaround (<10 min) + Battery powered	− Lower sensitivity and specificity in men, smokers and people with a high Body Mass Index − Requires internet access − Cannot determine drug sensitivity − Efficacy in LTBI and immunocompromised yet to be established
Raman spectroscopy	Active TB: 84.62%^10^ LTBI: 86.84%^10^	Active TB: 89.47%^10^ LTBI: 65%^10^	+ Portable + Battery powered + Low cost per sample (<$7) + Quick turnaround (<2 h) + Blood sample	− No efficacy established for HIV/Immunocompromised and paediatric patients − Small sample size in cited paper (*n* = 118) − No analysis of drug-resistance
AI Processing	68–96%^11^ (95% CI)	72–85%^11^ (95% CI)	+ Can be analysed on remote servers + Non-invasive + Diminishes human error	− Requires mobile phone data access − Preliminary results vary between populations
Arginine film	NA	NA	+ Quick turnaround (<2 h) + Culture-free + PCR free + Reported sensitivity down to 4.4 nM of DNA^12^	− Requires sputum sample − Requires un interruptible electricity supply

1 Yang J, Han X, Liu A, Bai X, Xu C, Bao F, et al. Use of Digital Droplet PCR to Detect Mycobacterium tuberculosis DNA in Whole Blood-Derived DNA Samples from Patients with Pulmonary and Extrapulmonary Tuberculosis. Front Cell Infect Microbiol. 2017;7:369;

2 Ai J-W, Zhou X, Xu T, Yang M, Chen Y, He G-Q, et al. CRISPR-based rapid and ultra-sensitive diagnostic test for Mycobacterium tuberculosis. Emerging Microbes & Infections. 2019;8(1):1361-9;

3 Ngabonziza JCS, Decroo T, Migambi P, Habimana YM, Van Deun A, Meehan CJ, et al. Prevalence and drivers of false-positive rifampicin-resistant Xpert MTB/RIF results: a prospective observational study in Rwanda. The Lancet Microbe. 2020;1(2):e74-e83;

4 Genoscreen Deeplex Myc-TB. Deeplex Myc-TB: From clinical samples to drug resistance profiles Lille, France: Genoscreen; 2020 [cited 2020 14/08]. Available from: https://www.genoscreen.fr/images/genoscreen-services/deeplex/technical_note_20200706_CE.pdf;

5 Makhado NA, Matabane E, Faccin M, Pinçon C, Jouet A, Boutachkourt F, et al. Outbreak of multidrug-resistant tuberculosis in South Africa undetected by WHO-endorsed commercial tests: an observational study. The Lancet Infectious Diseases. 2018;18(12):1350-9;

6 Tafess K, Ng TTL, Lao HY, Leung KSS, Tam KKG, Rajwani R, et al. Targeted-Sequencing Workflows for Comprehensive Drug Resistance Profiling of Mycobacterium tuberculosis Cultures Using Two Commercial Sequencing Platforms: Comparison of Analytical and Diagnostic Performance, Turnaround Time, and Cost. Clinical Chemistry. 2020;66(6):809-20;

7 Hoenen T, Groseth A, Rosenke K, Fischer RJ, Hoenen A, Judson SD, et al. Nanopore Sequencing as a Rapidly Deployable Ebola Outbreak Tool. Emerg Infect Dis. 2016;22(2):331-4;

8 Gupta RK, Turner CT, Venturini C, Esmail H, Rangaka MX, Copas A, et al. Concise whole blood transcriptional signatures for incipient tuberculosis: a systematic review and patient-level pooled meta-analysis. The Lancet Respiratory Medicine. 2020;8(4):395-406;

9 Saktiawati AMI, Stienstra Y, Subronto YW, Rintiswati N, Sumardi, Gerritsen J-W, et al. Sensitivity and specificity of an electronic nose in diagnosing pulmonary tuberculosis among patients with suspected tuberculosis. PLOS ONE. 2019;14(6):e0217963;

10 Kaewseekhao B, Nuntawong N, Eiamchai P, Roytrakul S, Reechaipichitkul W, Faksri K. Diagnosis of active tuberculosis and latent tuberculosis infection based on Raman spectroscopy and surface-enhanced Raman spectroscopy. Tuberculosis. 2020;121:101916;

11 Qin ZZ, Sander MS, Rai B, Titahong CN, Sudrungrot S, Laah SN, et al. Using artificial intelligence to read chest radiographs for tuberculosis detection: A multi-site evaluation of the diagnostic accuracy of three deep learning systems. Sci Rep. 2019;9(1):15000;

12 Eloi P, Nascimento GA, Córdula C, Visani V, Castelletti H, Bezerra G, et al. Toward a point-of-care diagnostic for specific detection of Mycobacterium tuberculosis from sputum samples. Tuberculosis 2020;121:101919.

i NA = Not available

ii CI = Confidence interval

## Conclusions

Despite the considerable scientific advances, tuberculosis remains inadequately diagnosed and monitored. Issues persist relating to insufficient diagnosis of latent cases as well as the worrying emergence of drug resistance. This is exacerbated by the obstacles of correctly identifying various (sub)-patient groups and co-morbidities whilst ensuring testing can be performed in the more rural areas of low- and middle-income countries. In the past decade, developments have been sought to overcome these challenges by for instance, reducing the reliance on electricity or by simplifying the testing procedures. In the chase of ‘new weapons’ to battle this old disease, many approaches are continually being developed, which are likely to change the landscape of tuberculosis diagnostics. However, none of these have yet reached their full potential and further technological developments are essential to optimise, test, and validate the methods highlighted in this review. Addressing these will further require considerable investments to fully develop and translate these diagnostic methods and propel their successful uptake to the global market. Whilst the scientific progress is making great strides forward in developing sophisticated diagnostic tools for TB, it is critical to maintain awareness and utilise TB monitoring programs as well as to continue the push for cooperation between the HIV and TB care, collectively aimed to mitigate the detrimental effects of this burdensome disease.

## Summary

- Despite significant advances, tuberculosis continues to affect millions of people worldwide annually.
- Early and effective rapid diagnosis plays a significant role in combatting TB.
- Many obstacles exist in identifying patients in rural areas, especially in low- and middle-income countries, exacerbated by limitations in current diagnostic methods.
- Ongoing research and advancements are being sought to overcome these obstacles and limitations using innovative emerging technologies.
- While these modern developments show promise in resolving many of the issues in TB diagnostics, they require investments and time to reach their full potential and enter the global markets. Whilst the scientific progress is making great strides forward, it is critical to maintain vigilance and continue utilising TB monitoring programs to continue the battle to defeat this ancient disease.

## Notes

1 Disease caused by Mycobacterium tuberculosis strains with resistance to, at least, isoniazid and rifampicin [1].

2 MDR-TB plus resistance to at least one of the fluoroquinolones and one of the injectable agents used in MDR-TB treatment regimens [1].

3 Defined by the WHO as *‘respiratory rate >30 breaths per min, body temperature >39°C, heart rate >120 beats per min, or unable to walk unaided'*

i NA = Not available

ii CI = Confidence interval

## Open Access

Open access for this article was enabled by the participation of University of Birmingham in an all-inclusive *Read & Publish* pilot with Portland Press and the Biochemical Society under a transformative agreement with JISC.

## Abbreviations

AI: Artificial Intelligence
DST: drug sensitivity testing
HIV: Human Immunodeficiency Virus
LAMP: loop-mediated isothermal amplification
LTBI: latent tuberculosis infection
MDR-TB: multi-drug resistant TB
MGIT: Mycobacteria growth indicator tubes
*Mtb*: *Mycobacterium tuberculosis*
NGS: next-generation sequencing
PCR: polymerase chain reaction
RPA: recombinase polymerase amplification
RS: Raman spectroscopy
TB: tuberculosis
WGS: whole-genome sequencing
WHO: World Health Organisation

## References

[1] Wirth T., Hildebrand F., Allix-Béguec C., Wölbeling F., Kubica T., Kremer K. et al. (2008) Origin, spread and demography of the *Mycobacterium tuberculosis* complex. PLoS Pathog. 4, e1000160 10.1371/journal.ppat.1000160 18802459 PMC2528947

[2] World Health Organization. (2020) Global Tuberculosis Report, World Health Organization, Geneva, Switzerland

[3] Bottai D., Frigui W., Sayes F., Di Luca M., Spadoni D., Pawlik A. et al. (2020) Tbd1 deletion as a driver of the evolutionary success of modern epidemic *Mycobacterium tuberculosis* lineages. Nat. Commun. 11, 684 10.1038/s41467-020-14508-5 32019932 PMC7000671

[4] Hunter R.L. (2018) The pathogenesis of tuberculosis: the early infiltrate of post-primary (Adult Pulmonary) tuberculosis: a distinct disease entity. Front. Immunol. 9, 2108 10.3389/fimmu.2018.02108 30283448 PMC6156532

[5] Spekker O., Hunt D.R., Paja L., Molnár E., Pálfi G. and Schultz M. (2020) Tracking down the White Plague: the skeletal evidence of tuberculous meningitis in the Robert J. Terry Anatomical Skeletal Collection. PLoS ONE 15, e0230418 10.1371/journal.pone.0230418 32187217 PMC7080279

[6] Tilahun M., Shibabaw A., Kiflie A., Bewket G., Abate E. and Gelaw B. (2019) Latent tuberculosis infection and associated risk factors among people living with HIV and apparently healthy blood donors at the University of Gondar referral hospital, Northwest Ethiopia. BMC Res. Notes 12, 515 10.1186/s13104-019-4548-x 31420007 PMC6698024

[7] National Institute for Health and Care Excellence. (2016) Tuberculosis (NG33), National Institute for Health and Care Excellence, London, U.K.

[8] Boeree M.J., Heinrich N., Aarnoutse R., Diacon A.H., Dawson R., Rehal S. et al. (2017) High-dose rifampicin, moxifloxacin, and SQ109 for treating tuberculosis: a multi-arm, multi-stage randomised controlled trial. Lancet Infect. Dis. 17, 39–49 10.1016/S1473-3099(16)30274-2 28100438 PMC5159618

[9] Yang T.W., Park H.O., Jang H.N., Yang J.H., Kim S.H., Moon S.H. et al. (2017) Side effects associated with the treatment of multidrug-resistant tuberculosis at a tuberculosis referral hospital in South Korea: a retrospective study. Medicine (Baltimore) 96, e7482 10.1097/MD.0000000000007482 28700490 PMC5515762

[10] Araújo-Mariz C., Lopes E.P., Acioli-Santos B., Maruza M., Montarroyos U.R., Ximenes R. et al. (2016) Hepatotoxicity during treatment for tuberculosis in people living with HIV/AIDS. PLoS ONE 11, e0157725 10.1371/journal.pone.0157725 27332812 PMC4917242

[11] Alemu A., Bitew Z.W. and Worku T. (2020) Poor treatment outcome and its predictors among drug-resistant tuberculosis patients in Ethiopia: a systematic review and meta-analysis. Int. J. Infect. Dis. 98, 420–439 10.1016/j.ijid.2020.05.087 32645375

[12] Dadu A., Hovhannesyan A., Ahmedov S., van der Werf M.J. and Dara M. (2020) Drug-resistant tuberculosis in Eastern Europe and Central Asia: a time-series analysis of routine surveillance data. Lancet Infect. Dis. 20, 250–258 10.1016/S1473-3099(19)30568-7 31784371

[13] Chisompola N.K., Streicher E.M., Muchemwa C.M.K., Warren R.M. and Sampson S.L. (2020) Molecular epidemiology of drug resistant *Mycobacterium tuberculosis* in Africa: a systematic review. BMC Infect. Dis. 20, 344 10.1186/s12879-020-05031-5 32404119 PMC7222473

[14] Tiberi S., Zumla A. and Migliori G.B. (2019) Multidrug and extensively drug-resistant tuberculosis: epidemiology, clinical features, management and treatment. Infect. Dis. Clin. North Am. 33, 1063–1085 10.1016/j.idc.2019.09.002 31668191

[15] Pang Y., Lu J., Huo F., Ma Y., Zhao L., Li Y. et al. (2017) Prevalence and treatment outcome of extensively drug-resistant tuberculosis plus additional drug resistance from the national clinical center for tuberculosis in China: a five-year review. J. Infect. 75, 433–440 10.1016/j.jinf.2017.08.005 28804028

[16] Sharma A., Hill A., Kurbatova E., van der Walt M., Kvasnovsky C., Tupasi T.E. et al. (2017) Estimating the future burden of multidrug-resistant and extensively drug-resistant tuberculosis in India, the Philippines, russia, and South Africa: a mathematical modelling study. Lancet Infect. Dis. 17, 707–715 10.1016/S1473-3099(17)30247-5 28499828 PMC5599934

[17] Wilson J.W. and Tsukayama D.T. (2016) Extensively drug-resistant tuberculosis: principles of resistance, diagnosis, and management. Mayo Clin. Proc. 91, 482–495 10.1016/j.mayocp.2016.01.014 26906649

[18] Shah N.S., Auld S.C., Brust J.C.M., Mathema B., Ismail N., Moodley P. et al. (2017) Transmission of extensively drug-resistant tuberculosis in South Africa. N. Engl. J. Med. 376, 243–253 10.1056/NEJMoa1604544 28099825 PMC5330208

[19] Kharsany A.B.M., McKinnon L.R., Lewis L., Cawood C., Khanyile D., Maseko D.V. et al. (2020) Population prevalence of sexually transmitted infections in a high HIV burden district in kwaZulu-Natal, South Africa: Implications for HIV epidemic control. Int. J. Infect. Dis. 98, 130–137 10.1016/j.ijid.2020.06.046 32562845 PMC7484252

[20] Sharan R., Bucşan A.N., Ganatra S., Paiardini M., Mohan M., Mehra S. et al. (2020) Chronic immune activation in TB/HIV co-infection. Trends Microbiol. 28, 619–632 10.1016/j.tim.2020.03.015 32417227 PMC7390597

[21] Mohammed H., Assefa N. and Mengistie B. (2018) Prevalence of extrapulmonary tuberculosis among people living with HIV/AIDS in sub-Saharan Africa: a systemic review and meta-analysis. HIV AIDS (Auckl) 10, 225–237 10.2147/HIV.S176587 30464643 PMC6225852

[22] Petruccioli E., Chiacchio T., Navarra A., Vanini V., Cuzzi G., Cimaglia C. et al. (2020) Effect of HIV-infection on QuantiFERON-plus accuracy in patients with active tuberculosis and latent infection. J. Infect. 80, 536–546 10.1016/j.jinf.2020.02.009 32097688 PMC8862140

[23] Nkengasong J.N., Mbopi-Keou F.-X., Peeling R.W., Yao K., Zeh C.E., Schneidman M. et al. (2018) Laboratory medicine in Africa since 2008: then, now, and the future. Lancet Infect. Dis. 18, e362–e367 10.1016/S1473-3099(18)30120-8 29980383 PMC13081755

[24] Lee J.Y. (2015) Diagnosis and treatment of extrapulmonary tuberculosis. Tuberc. Respir. Dis. (Seoul) 78, 47–55 10.4046/trd.2015.78.2.47 25861336 PMC4388900

[25] Zhou G., Luo Q., Luo S., Teng Z., Ji Z., Yang J. et al. (2020) Interferon-γ release assays or tuberculin skin test for detection and management of latent tuberculosis infection: a systematic review and meta-analysis. Lancet Infect. Dis. 10.1016/S1473-3099(20)30276-0 32673595

[26] Du J., Shu W., Liu Y., Wang Y., Zhan Y., Yu K. et al. (2019) Development and validation of external quality assessment panels for mycobacterial culture testing to diagnose tuberculosis in China. Eur. J. Clin. Microbiol. Infect. Dis. 38, 1961–1968 10.1007/s10096-019-03634-8 31342215

[27] Feng Z., Bai X., Wang T., Garcia C., Bai A., Li L. et al. (2020) Differential responses by human macrophages to infection with *Mycobacterium tuberculosis* and non-tuberculous mycobacteria. Front. Microbiol. 11, 116 10.3389/fmicb.2020.00116 32117140 PMC7018682

[28] Ma Y., Fan J., Li S., Dong L., Li Y., Wang F. et al. (2020) Comparison of lowenstein-Jensen medium and MGIT culture system for recovery of *Mycobacterium tuberculosis* from abscess samples. Diagn. Microbiol. Infect. Dis. 96, 114969 10.1016/j.diagmicrobio.2019.114969 31973887

[29] Kenaope L., Ferreira H., Seedat F., Otwombe K., Martinson N.A. and Variava E. (2020) Sputum culture and drug sensitivity testing outcome among X-pert *Mycobacterium tuberculosis*/rifampicin-positive, rifampicin-resistant sputum: A retrospective study: not all rifampicin resistance is multi-drug resistant. J. Glob. Antimicrob. Resist. 21, 434–438 10.1016/j.jgar.2019.11.008 31733411

[30] Nambiar R., Chatellier S., Bereksi N., van Belkum A., Singh N., Barua B. et al. (2017) Evaluation of Mycotube, a modified version of Lowenstein-Jensen (LJ) medium, for efficient recovery of *Mycobacterium tuberculosis* (MTB). Eur. J. Clin. Microbiol. Infect. Dis. 36, 1981–1988 10.1007/s10096-017-3052-2 28685188

[31] Advisory Committee on Dangerous Pathogens. (2013) The Approved List of biological agents In Department for Environment Food & Rural Affairs AaPHA (Department of Health and Social Care, ed.), 3rd edn, HM Government, London, U.K.

[32] Maehira Y. and Spencer R.C. (2019) Harmonization of biosafety and biosecurity standards for high-containment facilities in low- and middle-income countries: an approach from the perspective of occupational safety and health. Front. Public Health 7, 249 10.3389/fpubh.2019.00249 31572701 PMC6751378

[33] Ahmad M., Ibrahim W.H., Sarafandi S.A., Shahzada K.S., Ahmed S., Haq I.U. et al. (2019) Diagnostic value of bronchoalveolar lavage in the subset of patients with negative sputum/smear and mycobacterial culture and a suspicion of pulmonary tuberculosis. Int. J. Infect. Dis. 82, 96–101 10.1016/j.ijid.2019.03.021 30904678

[34] Kim Y.W., Kwon B.S., Lim S.Y., Lee Y.J., Cho Y.J., Yoon H.I. et al. (2020) Diagnostic value of bronchoalveolar lavage and bronchial washing in sputum-scarce or smear-negative cases with suspected pulmonary tuberculosis: a randomized study. Clin. Microbiol. Infect. 26, 911–916 10.1016/j.cmi.2019.11.013 31759097

[35] Neves C.P., Costa A.G., Safe I.P., de Souza Brito A., Jesus J.S., Kritski A.L. et al. (2020) The role of mini-bronchoalveolar lavage fluid in the diagnosis of pulmonary tuberculosis in critically ill patients. BMC Infect. Dis. 20, 229 10.1186/s12879-020-04954-3 32188399 PMC7081705

[36] Chadha V.K., Anjinappa S.M., Rade K., Baskaran D., Narang P., Kolappan C. et al. (2019) Sensitivity and specificity of screening tools and smear microscopy in active tuberculosis case finding. Ind. J. Tuberculosis 66, 99–104 10.1016/j.ijtb.2018.05.015 30797292

[37] Kunkel A., Abel Zur Wiesch P., Nathavitharana R.R., Marx F.M., Jenkins H.E. and Cohen T. (2016) Smear positivity in paediatric and adult tuberculosis: systematic review and meta-analysis. BMC Infect. Dis. 16, 282 10.1186/s12879-016-1617-9 27296716 PMC4906576

[38] Getahun H., Gunneberg C., Granich R. and Nunn P. (2010) HIV infection—associated tuberculosis: the epidemiology and the response. Clin. Infect. Dis. 50, S201–S207 10.1086/651492 20397949

[39] Park J.H., Choe J., Bae M., Choi S., Jung K.H., Kim M.J. et al. (2019) Clinical characteristics and radiologic features of immunocompromised patients with Pauci-bacillary pulmonary tuberculosis receiving delayed diagnosis and treatment. Open Forum. Infect. Dis. 6, ofz002 10.1093/ofid/ofz002 30775402 PMC6366656

[40] Ruhwald M., Aggerbeck H., Gallardo R.V., Hoff S.T., Villate J.I., Borregaard B. et al. (2017) Safety and efficacy of the C-Tb skin test to diagnose *Mycobacterium tuberculosis* infection, compared with an interferon γ release assay and the tuberculin skin test: a phase 3, double-blind, randomised, controlled trial. Lancet Respir. Med. 5, 259–268 10.1016/S2213-2600(16)30436-2 28159608

[41] Saktiawati A.M.I., Subronto Y.W., Stienstra Y., Sumardi, Supit F. and van der Werf T.S. (2019) Sensitivity and specificity of routine diagnostic work-up for tuberculosis in lung clinics in yogyakarta, Indonesia: a cohort study. BMC Public Health 19, 363 10.1186/s12889-019-6658-8 30940123 PMC6444523

[42] Qin Z.Z., Sander M.S., Rai B., Titahong C.N., Sudrungrot S., Laah S.N. et al. (2019) Using artificial intelligence to read chest radiographs for tuberculosis detection: A multi-site evaluation of the diagnostic accuracy of three deep learning systems. Sci. Rep. 9, 15000 10.1038/s41598-019-51503-3 31628424 PMC6802077

[43] Paton N.I., Borand L., Benedicto J., Kyi M.M., Mahmud A.M., Norazmi M.N. et al. (2019) Diagnosis and management of latent tuberculosis infection in Asia: review of current status and challenges. Int. J. Infect. Dis. 87, 21–29 10.1016/j.ijid.2019.07.004 31301458

[44] Toonkomdang S., Phinyo P., Phetsuksiri B., Patumanond J., Rudeeaneksin J. and Klayut W. (2020) Pragmatic accuracy of an in-house loop-mediated isothermal amplification (LAMP) for diagnosis of pulmonary tuberculosis in a Thai community hospital. PLoS ONE 15, e0236496 10.1371/journal.pone.0236496 32702008 PMC7377475

[45] Gelaw B., Shiferaw Y., Alemayehu M. and Bashaw A.A. (2017) Comparison of loop-mediated isothermal amplification assay and smear microscopy with culture for the diagnostic accuracy of tuberculosis. BMC Infect Dis. 17, 79 10.1186/s12879-016-2140-8 28095790 PMC5240421

[46] Singh B.K., Sharma S.K., Sharma R., Sreenivas V., Myneedu V.P., Kohli M. et al. (2017) Diagnostic utility of a line probe assay for multidrug resistant-TB in smear-negative pulmonary tuberculosis. PLoS ONE 12, e0182988 10.1371/journal.pone.0182988 28829779 PMC5568731

[47] Bjerrum S., Schiller I., Dendukuri N., Kohli M., Nathavitharana R.R., Zwerling A.A. et al. (2019) Lateral flow urine lipoarabinomannan assay for detecting active tuberculosis in people living with HIV. Cochrane Database Syst. Rev. 10, CD011420 10.1002/14651858.CD011420.pub3 31633805 PMC6802713

[48] Ayubi E., Doosti-Irani A., Sanjari Moghaddam A., Sani M., Nazarzadeh M. and Mostafavi E. (2016) The clinical usefulness of tuberculin skin test versus interferon-gamma release assays for diagnosis of latent tuberculosis in HIV patients: a meta-analysis. PLoS ONE 11, e0161983 10.1371/journal.pone.0161983 27622293 PMC5021339

[49] Benachinmardi K.K., Sangeetha S., Rao M. and Prema R. (2019) Validation and clinical application of interferon-gamma release assay for diagnosis of latent tuberculosis infection in children. Int. J. Appl. Basic Med. Res. 9, 241–245 10.4103/ijabmr.IJABMR_86_19 31681551 PMC6822318

[50] Subbaraman R., Nathavitharana R.R., Satyanarayana S., Pai M., Thomas B.E., Chadha V.K. et al. (2016) The tuberculosis cascade of care in India's public sector: a systematic review and meta-analysis. PLoS Med. 13, e1002149 10.1371/journal.pmed.1002149 27780217 PMC5079571

[51] Nyaruaba R., Mwaliko C., Kering K.K. and Wei H. (2019) Droplet digital PCR applications in the tuberculosis world. Tuberculosis 117, 85–92 10.1016/j.tube.2019.07.001 31378274

[52] Rigouts L., Miotto P., Schats M., Lempens P., Cabibbe A.M., Galbiati S. et al. (2019) Fluoroquinolone heteroresistance in *Mycobacterium tuberculosis*: detection by genotypic and phenotypic assays in experimentally mixed populations. Sci. Rep. 9, 11760 10.1038/s41598-019-48289-9 31409849 PMC6692311

[53] Luo J., Luo M., Li J., Yu J., Yang H., Yi X. et al. (2019) Rapid direct drug susceptibility testing of *mycobacterium tuberculosis* based on culture droplet digital polymerase chain reaction. Int. J. Tuberc. Lung Dis. 23, 219–225 10.5588/ijtld.18.0182 30808455

[54] Yang J., Han X., Liu A., Bai X., Xu C., Bao F. et al. (2017) Use of digital droplet PCR to detect *Mycobacterium tuberculosis* DNA in whole blood-derived DNA samples from patients with pulmonary and extrapulmonary tuberculosis. Front. Cell Infect. Microbiol. 7, 369 10.3389/fcimb.2017.00369 28848722 PMC5554497

[55] Kuypers J. and Jerome K.R. (2017) Applications of digital PCR for clinical microbiology. J. Clin. Microbiol. 55, 1621–1628 10.1128/JCM.00211-17 28298452 PMC5442518

[56] Li S.-Y., Cheng Q.-X., Liu J.-K., Nie X.-Q., Zhao G.-P. and Wang J. (2018) CRISPR-Cas12a has both cis- and trans-cleavage activities on single-stranded DNA. Cell Res. 28, 491–493 10.1038/s41422-018-0022-x 29531313 PMC5939048

[57] Ai J.-W., Zhou X., Xu T., Yang M., Chen Y., He G.-Q. et al. (2019) CRISPR-based rapid and ultra-sensitive diagnostic test for *Mycobacterium tuberculosis*. Emerg. Microbes Infect. 8, 1361–1369 10.1080/22221751.2019.1664939 31522608 PMC6758691

[58] Gootenberg J.S., Abudayyeh O.O., Kellner M.J., Joung J., Collins J.J. and Zhang F. (2018) Multiplexed and portable nucleic acid detection platform with Cas13, Cas12a, and Csm6. Science 360, 439–444 10.1126/science.aaq0179 29449508 PMC5961727

[59] Mukama O., Wu J., Li Z., Liang Q., Yi Z., Lu X. et al. (2020) An ultrasensitive and specific point-of-care CRISPR/Cas12 based lateral flow biosensor for the rapid detection of nucleic acids. Biosens. Bioelectron. 159, 112143 10.1016/j.bios.2020.112143 32364943

[60] Wang S., Li H., Kou Z., Ren F., Jin Y., Yang L. et al. (2020) Highly sensitive and specific detection of hepatitis B virus DNA and drug resistance mutations utilizing the PCR-based CRISPR-Cas13a system. Clin. Microbiol. Infect. 10.1016/j.cmi.2020.04.018 32360447

[61] Genoscreen Deeplex Myc-TB. (2020) Deeplex Myc-TB: From clinical samples to drug resistance profiles Lille, France: Genoscreen; [cited 2020 14/08]. Available from: https://www.genoscreen.fr/images/genoscreen-services/deeplex/technical_note_20200706_CE.pdf

[62] World Health Organization. (2018) The use of Next-Generation Sequencing Technologies for the Detection of Mutations Associated with Drug Resistance in Mycobacterium Tuberculosis Complex: Technical Guide, World Health Organization, Geneva, Switzerland

[63] Kavvas E.S., Catoiu E., Mih N., Yurkovich J.T., Seif Y., Dillon N. et al. (2018) Machine learning and structural analysis of *Mycobacterium tuberculosis* pan-genome identifies genetic signatures of antibiotic resistance. Nat. Commun. 9, 4306 10.1038/s41467-018-06634-y 30333483 PMC6193043

[64] Brown A.C., Bryant J.M., Einer-Jensen K., Holdstock J., Houniet D.T., Chan J.Z.M. et al. (2015) Rapid whole-genome sequencing of *Mycobacterium tuberculosis* isolates directly from clinical samples. J. Clin. Microbiol. 53, 2230–2237 10.1128/JCM.00486-15 25972414 PMC4473240

[65] Quick J., Loman N.J., Duraffour S., Simpson J.T., Severi E., Cowley L. et al. (2016) Real-time, portable genome sequencing for Ebola surveillance. Nature 530, 228–232 10.1038/nature16996 26840485 PMC4817224

[66] Cui J.-Y., Liang H.-W., Pan X.-L., Li D., Jiao N., Liu Y.-H. et al. (2017) Characterization of a novel panel of plasma microRNAs that discriminates between *mycobacterium tuberculosis* infection and healthy individuals. PLoS ONE 12, e0184113 10.1371/journal.pone.0184113 28910318 PMC5598944

[67] Ndzi E.N., Nkenfou C.N., Mekue L.M., Zentilin L., Tamgue O., Pefura E.W.Y. et al. (2019) MicroRNA hsa-miR-29a-3p is a plasma biomarker for the differential diagnosis and monitoring of tuberculosis. Tuberculosis 114, 69–76 10.1016/j.tube.2018.12.001 30711160

[68] Correia C.N., Nalpas N.C., McLoughlin K.E., Browne J.A., Gordon S.V., MacHugh D.E. et al. (2017) Circulating microRNAs as potential biomarkers of infectious disease. Front. Immunol. 8, 118 10.3389/fimmu.2017.00118 28261201 PMC5311051

[69] Togun T.O., MacLean E., Kampmann B. and Pai M. (2018) Biomarkers for diagnosis of childhood tuberculosis: a systematic review. PLoS ONE 13, e0204029 10.1371/journal.pone.0204029 30212540 PMC6136789

[70] Saktiawati A.M.I., Stienstra Y., Subronto Y.W., Rintiswati N., Sumardi, Gerritsen J.W. et al. (2019) Sensitivity and specificity of an electronic nose in diagnosing pulmonary tuberculosis among patients with suspected tuberculosis. PLoS ONE 14, e0217963 10.1371/journal.pone.0217963 31194793 PMC6563983

[71] Saktiawati A.M.I., Putera D.D., Setyawan A., Mahendradhata Y. and van der Werf T.S. (2019) Diagnosis of tuberculosis through breath test: a systematic review. EBioMedicine 46, 202–214 10.1016/j.ebiom.2019.07.056 31401197 PMC6712009

[72] Lorenz B., Wichmann C., Stöckel S., Rösch P. and Popp J. (2017) Cultivation-free Raman spectroscopic investigations of bacteria. Trends Microbiol. 25, 413–424 10.1016/j.tim.2017.01.002 28188076

[73] Kaewseekhao B., Nuntawong N., Eiamchai P., Roytrakul S., Reechaipichitkul W. and Faksri K. (2020) Diagnosis of active tuberculosis and latent tuberculosis infection based on Raman spectroscopy and surface-enhanced Raman spectroscopy. Tuberculosis 121, 101916 10.1016/j.tube.2020.101916 32279876

[74] Mühlig A., Bocklitz T., Labugger I., Dees S., Henk S., Richter E. et al. (2016) LOC-SERS: a promising closed system for the identification of *mycobacteria*. Anal. Chem. 88, 7998–8004 10.1021/acs.analchem.6b01152 27441738

[75] Stöckel S., Meisel S., Lorenz B., Kloß S., Henk S., Dees S. et al. (2017) Raman spectroscopic identification of *Mycobacterium tuberculosis*. J. Biophotonics 10, 727–734 10.1002/jbio.201600174 27714969

[76] Shah M.I., Mishra S., Yadav V.K., Chauhan A., Sarkar M., Sharma S.K. et al. (2017) Ziehl-Neelsen sputum smear microscopy image database: a resource to facilitate automated bacilli detection for tuberculosis diagnosis. J. Med. Imaging (Bellingham) 4, 027503 10.1117/1.JMI.4.2.027503 28680911 PMC5492794

[77] Harris M., Qi A., Jeagal L., Torabi N., Menzies D., Korobitsyn A. et al. (2019) A systematic review of the diagnostic accuracy of artificial intelligence-based computer programs to analyze chest x-rays for pulmonary tuberculosis. PLoS ONE 14, e0221339 10.1371/journal.pone.0221339 31479448 PMC6719854

[78] Wahl B., Cossy-Gantner A., Germann S. and Schwalbe N.R. (2018) Artificial intelligence (AI) and global health: how can AI contribute to health in resource-poor settings? BMJ Glob. Health 3, e000798 10.1136/bmjgh-2018-000798 PMC6135465 30233828

[79] Eloi P., Nascimento G.A., Córdula C., Visani V., Castelletti H., Bezerra G. et al. (2020) Toward a point-of-care diagnostic for specific detection of *Mycobacterium tuberculosis* from sputum samples. Tuberculosis 121, 101919 10.1016/j.tube.2020.101919 32279875

[80] Jaroenram W., Kampeera J., Arunrut N., Karuwan C., Sappat A., Khumwan P. et al. (2020) Graphene-based electrochemical genosensor incorporated loop-mediated isothermal amplification for rapid on-site detection of *Mycobacterium tuberculosis*. J. Pharm. Biomed. Anal. 186, 113333 10.1016/j.jpba.2020.113333 32402994

